# Crystal structure of a new monoclinic polymorph of 2,4-di­hydroxy­benzaldehyde 4-methyl­thio­semi­carbazone

**DOI:** 10.1107/S2056989014026498

**Published:** 2015-01-01

**Authors:** M. A. Salam, Mouayed A. Hussein, Edward R. T. Tiekink

**Affiliations:** a Bangladesh Petroleum Exploration and Production Co. Ltd (BAPEX), 4 Karwan Bazar, BAPEX Bhabon, Dhaka 1215, Bangladesh; b Department of Chemistry, College of Science, University of Basrah, Basra 61004, Iraq; c Department of Chemistry, University of Malaya, 50603 Kuala Lumpur, Malaysia

**Keywords:** crystal structure, thio­semicarbazone, polymorph, conformation, hydrogen bonding

## Abstract

The title compound, C_9_H_11_N_3_O_2_S, is a second monoclinic (*P*2_1_/*c*) polymorph of the previously reported *Cc* form [Tan *et al.* (2008*b* ▸). *Acta Cryst.* E**64**, o2224]. The mol­ecule is non-planar, with the dihedral angle between the N_3_CS residue (r.m.s. deviation = 0.0816 Å) and the benzene ring being 21.36 (4)°. The conformation about the C=N bond [1.292 (2) Å] is *E*, the two N-bound H atoms are *anti*, and the inner hy­droxy O-bound and outer amide N-bound H atoms form intra­molecular hydrogen bonds to the imine N atom. Crucially, the H atom of the outer hy­droxy group is approximately *syn* to the H atom of the benzene C atom connecting the two C atoms bearing the hy­droxy substituents. This arrangement enables the formation of supra­molecular tubes aligned along [010] and sustained by N—H⋯O, O—H⋯S and N—H⋯S hydrogen bonds; the tubes pack with no specific inter­actions between them. While the mol­ecular structure in the *Cc* form is comparable, the H atom of the outer hy­droxy group is approximately *anti*, rather than *syn*. This different orientation leads to the formation a three-dimensional architecture based on N—H⋯O and O—H⋯S hydrogen bonds.

## Abstract 2

A new monoclinic (*P*2_1_/*c*) polymorph of the title compound has the same overall conformation as a previously reported (*Cc*) form with the exception of the conformation of the outer hy­droxy H atom. This difference results in very different crystal packing based on hydrogen bonding, *i.e.* supra­molecular tubes in the new form as opposed to a three-dimensional architecture in the *Cc* form.

## Chemical context   

In a review of the biological applications of metal complexes of thio­semicarbazone derivatives, Dilworth & Hueting (2012 ▸) highlighted the various biological roles exhibited by this class of compound. Thus, these may have therapeutic potential, for example being cytotoxic and capable of inhibiting both ribonuclease reductase and topoisomerase II. Metal complexes of thio­semicarbazones can also function as diagnostic agents in imaging/diagnostic applications. In the context of this bio­logical relevance, the specific title compound of the present report has been coordinated as an *N*,*O*,*S*-tridentate dianion to zinc(II) and the resultant complex explored for activity against prostate cancer (Tan *et al.*, 2012 ▸).
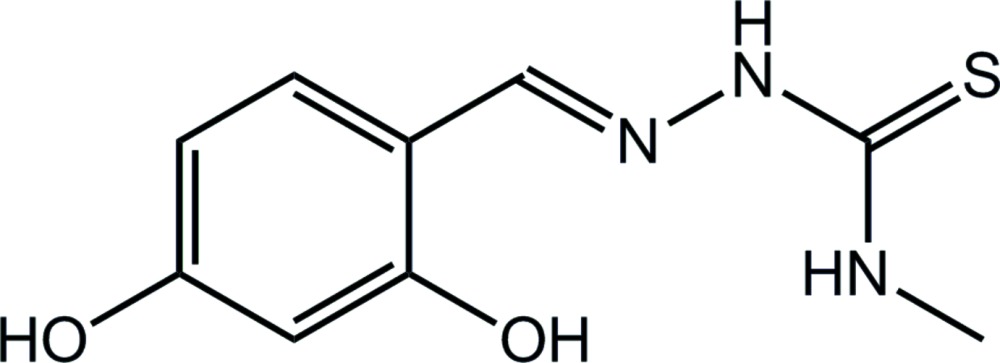


The crystal structure of the title mol­ecule has been reported previously as a *Cc* polymorph (Tan *et al.*, 2008*b* ▸). Following on from previous structural work on related compounds (Affan *et al.*, 2013 ▸), the title compound was prepared and routine screening of the crystals indicated that this crystallizes as a second monoclinic (*P*2_1_/*c*) polymorph. The crystal and mol­ecular structure of the second form of the title compound is reported herein and compared with the original *Cc* polymorph.

## Structural commentary   

**Figure 1:**
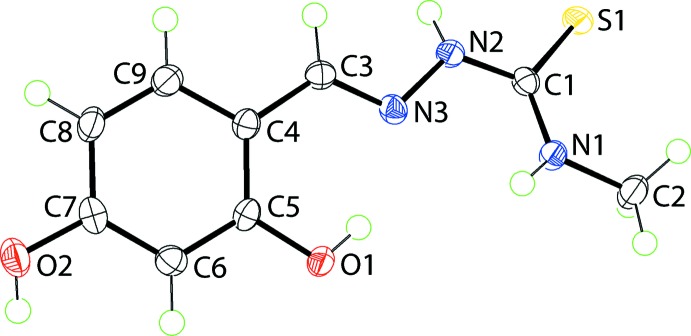
The mol­ecular structure of the title compound in the *P*2_1_/*c* polymorph, showing the atom labelling and displacement ellipsoids at the 70% probability level.

The mol­ecular structure found in the new monoclinic (*P*2_1_/*c*) polymorph is shown in Fig. 1 ▸. The mol­ecule is non-planar with a twist about the C1—N2 bond being evident as seen in (i) the N3—N2—C1—S1 torsion angle of 164.83 (11)° and (ii) the dihedral angle between the N_3_CS residue (r.m.s. deviation = 0.0816 Å) and benzene ring of 21.36 (4)°. The conformation about the C3=N3 bond [1.292 (2) Å] is *E*, the two N-bound H atoms are *anti*, and within the mol­ecule, both the O1- and N1-bound H atoms form intra­molecular hydrogen bonds to the imine-N3 atom, Table 1 ▸. The O2—H2*o* H atom is approximately *syn* to the C6—H6 H atom.

**Figure 2:**
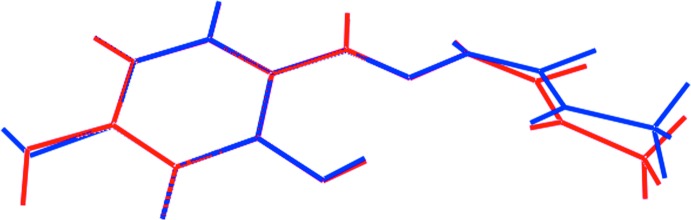
Overlay diagram of the mol­ecules in the *P*2_1_/*n* polymorph (red image) and in the *Cc* form (blue). The mol­ecules have been overlapped so the benzene rings are coincident.

To a first approximation, the mol­ecular structure found in the *Cc* polymorph (Tan *et al.*, 2008*b* ▸), reported to be isolated also from an ethanol solution, is similar, but two significant differences are noted. These are highlighted in the overlay diagram shown in Fig. 2 ▸. With the N3—N2—C1—S1 torsion angle being −172.5 (2)°, the twist about the C1—N2 bond deviates by about 8°, toward planarity, from that in the *P*2_1_/*c* form. However, the dihedral angle between the N_3_CS residue and benzene ring of 23.1 (9)° is a little wider in the *Cc* form as the terminal methyl group is slightly twisted out of the CN_3_S plane: the C2—N1—C1—S1 torsion angle is −3.1 (5)° *cf*. to 1.2 (2)° in the *P*2_1_/*c* form. The major and most significant difference arises in the relative orientation of the outer hy­droxy group where the H2*o* atom is *anti* to the C6—H6 H atom *cf*. approximately *syn* in the *P*2_1_/*c* form. This has a major consequence upon the crystal packing in the two forms as discussed in §3.

The calculated density for the *P*2_1_/*c* form is 1.496 g cm^−3^ and the packing efficiency (KPI), calculated by *PLATON* (Spek, 2009 ▸), is 73.1%. These values are lower than the comparable values in the *Cc* form, *i.e*. 1.521 g cm^−3^ and 74.4%, respectively, suggesting that the *Cc* form is the more stable.

## Supra­molecular features   

**Figure 3:**
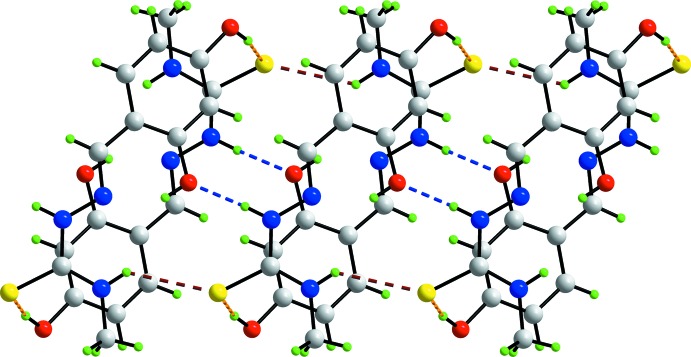
Supra­molecular tube along the *b* axis in the structure of the *P*2_1_/*c* polymorph sustained by N—H⋯O, O—H⋯S and N—H⋯S hydrogen bonds, shown as blue, orange and brown dashed lines, respectively (see Table 1 ▸ for details).

**Figure 4:**
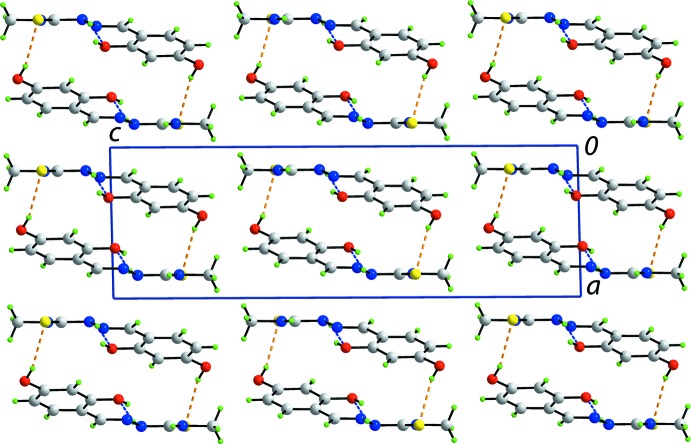
View in projection down the *b* axis of the unit-cell contents of the *P*2_1_/*c* polymorph, highlighting the packing of the supra­molecular tubes.

**Figure 5:**
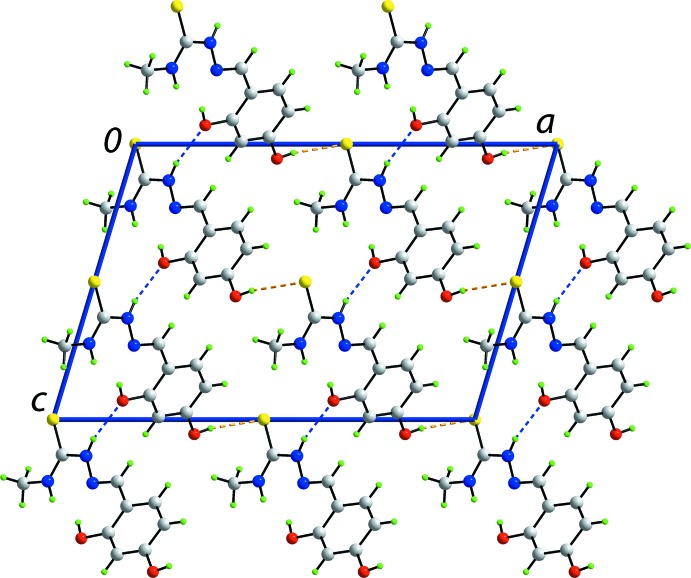
View in projection down the *b* axis of the unit-cell contents of the *Cc* polymorph, highlighting the the stacking of the layers along the *a* axis, sustained by N—H⋯O hydrogen bonds (blue dashed lines), and their connection by O—H⋯S hydrogen bonds (orange dashed lines).

In the crystal packing of the *P*2_1_/*c* polymorph, conventional hydrogen bonding inter­actions lead to the formation of a supra­molecular tube, Fig. 3 ▸ and Table 1 ▸. Here, the inner N2—H2*n* atom forms a hydrogen bond to a translationally related inner O1 atom, and the bifurcated S1 atom accepts hydrogen bonds from the outer, centrosymmetically related, O2—H2*o* and a translationally related, outer N1—H1*n* atom. The tubes are aligned along the *b* axis and pack with no specific inter­molecular inter­actions between them, Fig. 4 ▸. A distinctive crystal packing pattern is noted in the *Cc* polymorph (Tan *et al.*, 2008*b* ▸). Here, the inner N2—H2*n* atom forms a hydrogen bond to a glide-related inner O1 atom, leading to a supra­molecular layer that stacks along the *a* axis. The S1 atoms project to one side of the layer and the outer O2—H2*o* atoms, with the *anti* disposition (see above), lie to the other. These form hydrogen bonds so that a three-dimensional architecture ensues, Fig. 5 ▸. In this scenario, the outer N1—H1*n* atom only participates in an intra­molecular hydrogen bond to the N3 atom, as does in the inner O1—H1*o* atom.

## Database survey   

Given the inter­est in semi­thio­carbazones owing to their biological potential, it is not surprising that a search of Version 5.35 (plus May updates) of the Cambridge Crystallographic Database (Groom & Allen, 2014 ▸) revealed almost 100 hits for the CC(H)=NN(H)C(=S)N(H)C fragment. The only restriction in the search was that the heaviest atom be S. In the absence of this restriction there were nearly 400 hits. Of the smaller set of structures, there was only one pair of polymorphs, namely two triclinic (*P* 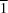) forms for salicyl­aldehyde 4-phenyl­thio­semicarbazone, one with *Z*′ = 3 (Seena *et al.*, 2008 ▸) and the other with *Z*′ = 2 (Rubčić *et al.*, 2008 ▸). The most closely related structure in the literature is the N-Et derivative, reported twice (Tan *et al.*, 2008*a* ▸; Hussein *et al.*, 2014 ▸). This structure exhibits the same mol­ecular attributes as described above for the N-Me polymorphs, *i.e*. conformation, relative disposition of key atoms and intra­molecular hydrogen bonding.

## Synthesis and crystallization   

A solution of 2,4-di­hydroxy­benzaldehyde (0.65 g, 4.75 mmol) in ethanol (20 ml) was added to a solution of 4-methyl-3-thio­semicarbazide (0.5 g, 4.75 mmol) in ethanol (20 ml). The resulting brown solution was refluxed with stirring for 2 h, and then filtered, washed with ethanol and dried *in vacuo* over silica gel. The filtrate was left to stand at room temperature for two days after which colourless block-like crystals were obtained (yield 0.79 g, 74%). M.p: 471–473 K. FT–IR (KBr, cm^−1^) ν_max_: 3377 (*s*, OH), 3190 (*s*, NH), 1615 (*m*, C=N), 1558 (*s*, C—O), 1012 (*m*, N—N), 1360, 845 (*w*, C=S). Analysis calculated for C_9_H_11_N_3_O_2_S: C, 47.94; H, 4.88; N, 18.64%. Found: C, 48.0; H, 4.68; N, 18.52%.

## Refinement   

Crystal data, data collection and structure refinement details are summarized in Table 2 ▸. Carbon-bound H-atoms were placed in calculated positions (C—H = 0.95–0.98 Å) and included in the refinement in the riding-model approximation, with *U*_iso_(H) =1.5*U*_eq_(C) for methyl H atoms and = 1.2*U*_eq_(C) for other H atoms. The O- and N-bound H-atoms were located in a difference Fourier map and freely refined.

## Supplementary Material

Crystal structure: contains datablock(s) I, global. DOI: 10.1107/S2056989014026498/su5033sup1.cif

Structure factors: contains datablock(s) I. DOI: 10.1107/S2056989014026498/su5033Isup2.hkl

Supporting information file. DOI: 10.1107/S2056989014026498/su5033Isup3.cml

CCDC reference: 960620

Additional supporting information:  crystallographic information; 3D view; checkCIF report

## Figures and Tables

**Table 1:** Hydrogen-bond geometry (, )

*D*H*A*	*D*H	H*A*	*D* *A*	*D*H*A*
O1H1*o*N3	0.83(2)	1.97(2)	2.6992(17)	147(2)
N1H1*n*N3	0.815(19)	2.35(2)	2.7080(19)	107.1(16)
O2H2*o*S1^i^	0.90(2)	2.37(2)	3.1918(12)	152(2)
N1H1*n*S1^ii^	0.815(19)	2.763(18)	3.3883(13)	134.9(17)
N2H2*n*O1^iii^	0.90(2)	2.08(2)	2.9527(17)	162(2)

Symmetry codes: (i) 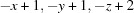; (ii) 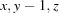; (iii) 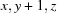.

**Table 2:** Experimental details

Crystal data	
Chemical formula	C_9_H_11_N_3_O_2_S
*M*_r_	225.27
Crystal system, space group	Monoclinic, *P*2_1_/*c*
Temperature (K)	100
*a*, *b*, *c* ()	7.3058(2), 6.0582(1), 22.6041(6)
()	91.100(2)
*V* (^3^)	1000.27(4)
*Z*	4
Radiation type	Mo *K*
(mm^1^)	0.31
Crystal size (mm)	0.48 0.19 0.14

Data collection	
Diffractometer	Bruker APEXII CCD
Absorption correction	Multi-scan (*SADABS*; Sheldrick, 1996 ▸)
*T*_min_, *T*_max_	0.866, 0.957
No. of measured, independent and observed [*I* > 2(*I*)] reflections	9696, 2302, 1950
*R*_int_	0.027
(sin /)_max_ (^1^)	0.650

Refinement	
*R*[*F* ^2^ > 2(*F* ^2^)], *wR*(*F* ^2^), *S*	0.035, 0.086, 1.06
No. of reflections	2302
No. of parameters	153
H-atom treatment	H atoms treated by a mixture of independent and constrained refinement
_max_, _min_ (e ^3^)	0.30, 0.31

Computer programs: *APEX2* and *SAINT* (Bruker, 2009 ▸), *SHELXS2014* and *SHELXL2014* (Sheldrick, 2008 ▸), *ORTEP-3 for Windows* (Farrugia, 2012 ▸), *QMol* (Gans Shalloway, 2001 ▸), *DIAMOND* (Brandenburg, 2006 ▸), *PLATON* (Spek, 2009 ▸ and *publCIF* (Westrip, 2010 ▸).

## supplementary crystallographic information

### Crystal data

**Table:**

C_9_H_11_N_3_O_2_S	*F*(000) = 472
*M**_r_* = 225.27	*D*_x_ = 1.496 Mg m^−^^3^
Monoclinic, *P*2_1_/*c*	Mo *K*α radiation, λ = 0.71073 Å
*a* = 7.3058 (2) Å	Cell parameters from 3917 reflections
*b* = 6.0582 (1) Å	θ = 3.3–29.8°
*c* = 22.6041 (6) Å	µ = 0.31 mm^−^^1^
β = 91.100 (2)°	*T* = 100 K
*V* = 1000.27 (4) Å^3^	Block, colourless
*Z* = 4	0.48 × 0.19 × 0.14 mm

### Data collection

**Table:**

Bruker APEXII CCD diffractometer	2302 independent reflections
Radiation source: sealed tube	1950 reflections with *I* > 2σ(*I*)
Graphite monochromator	*R*_int_ = 0.027
φ and ω scans	θ_max_ = 27.5°, θ_min_ = 1.8°
Absorption correction: multi-scan (*SADABS*; Sheldrick, 1996)	*h* = −9→9
*T*_min_ = 0.866, *T*_max_ = 0.957	*k* = −7→7
9696 measured reflections	*l* = −29→24

### Refinement

**Table:**

Refinement on *F*^2^	0 restraints
Least-squares matrix: full	Hydrogen site location: mixed
*R*[*F*^2^ > 2σ(*F*^2^)] = 0.035	H atoms treated by a mixture of independent and constrained refinement
*wR*(*F*^2^) = 0.086	*w* = 1/[σ^2^(*F*_o_^2^) + (0.0336*P*)^2^ + 0.7405*P*] where *P* = (*F*_o_^2^ + 2*F*_c_^2^)/3
*S* = 1.06	(Δ/σ)_max_ = 0.001
2302 reflections	Δρ_max_ = 0.30 e Å^−^^3^
153 parameters	Δρ_min_ = −0.31 e Å^−^^3^

### Special details

**Table:**

Geometry. All e.s.d.'s (except the e.s.d. in the dihedral angle between two l.s. planes) are estimated using the full covariance matrix. The cell e.s.d.'s are taken into account individually in the estimation of e.s.d.'s in distances, angles and torsion angles; correlations between e.s.d.'s in cell parameters are only used when they are defined by crystal symmetry. An approximate (isotropic) treatment of cell e.s.d.'s is used for estimating e.s.d.'s involving l.s. planes.

### Fractional atomic coordinates and isotropic or equivalent isotropic displacement parameters (Å^2^)

**Table:**

	*x*	*y*	*z*	*U*_iso_*/*U*_eq_	
S1	0.15271 (5)	1.11056 (7)	1.14702 (2)	0.01707 (12)	
O1	0.33247 (16)	0.2527 (2)	1.00557 (5)	0.0174 (3)	
H1o	0.301 (3)	0.362 (4)	1.0247 (11)	0.044 (7)*	
O2	0.45517 (16)	0.0527 (2)	0.80619 (5)	0.0204 (3)	
H2o	0.541 (3)	−0.033 (4)	0.8232 (11)	0.051 (7)*	
N1	0.15573 (18)	0.6695 (2)	1.14178 (6)	0.0148 (3)	
H1n	0.153 (3)	0.557 (3)	1.1220 (9)	0.019 (5)*	
N2	0.15295 (18)	0.8552 (2)	1.05291 (6)	0.0152 (3)	
H2n	0.183 (3)	0.984 (4)	1.0359 (9)	0.029 (5)*	
N3	0.19726 (17)	0.6617 (2)	1.02301 (6)	0.0139 (3)	
C1	0.1544 (2)	0.8599 (3)	1.11312 (7)	0.0135 (3)	
C2	0.1526 (2)	0.6471 (3)	1.20604 (7)	0.0197 (4)	
H2A	0.2699	0.6968	1.2231	0.030*	
H2B	0.1326	0.4920	1.2164	0.030*	
H2C	0.0534	0.7374	1.2217	0.030*	
C3	0.1997 (2)	0.6841 (3)	0.96619 (7)	0.0141 (3)	
H3	0.1592	0.8197	0.9494	0.017*	
C4	0.2616 (2)	0.5119 (3)	0.92679 (7)	0.0137 (3)	
C5	0.3302 (2)	0.3078 (3)	0.94674 (7)	0.0135 (3)	
C6	0.3978 (2)	0.1534 (3)	0.90734 (7)	0.0154 (3)	
H6	0.4461	0.0173	0.9214	0.019*	
C7	0.3941 (2)	0.1999 (3)	0.84699 (7)	0.0157 (3)	
C8	0.3264 (2)	0.4006 (3)	0.82584 (7)	0.0177 (3)	
H8	0.3239	0.4312	0.7846	0.021*	
C9	0.2630 (2)	0.5540 (3)	0.86554 (7)	0.0168 (3)	
H9	0.2192	0.6920	0.8512	0.020*	

### Atomic displacement parameters (Å^2^)

**Table:**

	*U*^11^	*U*^22^	*U*^33^	*U*^12^	*U*^13^	*U*^23^
S1	0.0239 (2)	0.0115 (2)	0.0158 (2)	0.00296 (15)	0.00059 (15)	−0.00209 (15)
O1	0.0274 (6)	0.0135 (6)	0.0114 (6)	0.0017 (5)	0.0029 (5)	0.0007 (5)
O2	0.0214 (6)	0.0243 (7)	0.0154 (6)	0.0041 (5)	0.0010 (5)	−0.0062 (5)
N1	0.0223 (7)	0.0099 (7)	0.0120 (7)	−0.0017 (5)	0.0015 (5)	−0.0019 (6)
N2	0.0215 (7)	0.0103 (7)	0.0137 (7)	0.0012 (5)	0.0019 (5)	−0.0007 (5)
N3	0.0163 (6)	0.0116 (6)	0.0139 (7)	−0.0005 (5)	0.0011 (5)	−0.0023 (5)
C1	0.0119 (7)	0.0145 (8)	0.0141 (8)	0.0005 (6)	0.0004 (6)	−0.0009 (6)
C2	0.0279 (9)	0.0177 (8)	0.0137 (8)	−0.0017 (7)	0.0020 (6)	0.0019 (7)
C3	0.0144 (7)	0.0125 (7)	0.0153 (8)	−0.0008 (6)	0.0001 (6)	0.0006 (6)
C4	0.0140 (7)	0.0143 (8)	0.0129 (7)	−0.0016 (6)	0.0011 (6)	0.0002 (6)
C5	0.0145 (7)	0.0150 (8)	0.0110 (7)	−0.0036 (6)	0.0004 (5)	0.0005 (6)
C6	0.0150 (7)	0.0136 (8)	0.0177 (8)	−0.0006 (6)	0.0018 (6)	0.0009 (6)
C7	0.0139 (7)	0.0180 (8)	0.0152 (8)	−0.0016 (6)	0.0025 (6)	−0.0043 (6)
C8	0.0184 (7)	0.0240 (9)	0.0106 (7)	0.0022 (6)	0.0006 (6)	0.0008 (7)
C9	0.0163 (7)	0.0187 (8)	0.0153 (8)	0.0008 (6)	0.0005 (6)	0.0027 (7)

### Geometric parameters (Å, º)

**Table:**

S1—C1	1.7011 (16)	C2—H2B	0.9800
O1—C5	1.3707 (19)	C2—H2C	0.9800
O1—H1o	0.83 (3)	C3—C4	1.449 (2)
O2—C7	1.3640 (19)	C3—H3	0.9500
O2—H2o	0.90 (3)	C4—C5	1.405 (2)
N1—C1	1.323 (2)	C4—C9	1.408 (2)
N1—C2	1.459 (2)	C5—C6	1.389 (2)
N1—H1n	0.81 (2)	C6—C7	1.393 (2)
N2—C1	1.361 (2)	C6—H6	0.9500
N2—N3	1.3945 (18)	C7—C8	1.394 (2)
N2—H2n	0.90 (2)	C8—C9	1.378 (2)
N3—C3	1.292 (2)	C8—H8	0.9500
C2—H2A	0.9800	C9—H9	0.9500
			
C5—O1—H1o	108.3 (17)	C4—C3—H3	118.5
C7—O2—H2o	108.9 (16)	C5—C4—C9	117.76 (14)
C1—N1—C2	124.63 (14)	C5—C4—C3	123.32 (14)
C1—N1—H1n	117.4 (14)	C9—C4—C3	118.82 (14)
C2—N1—H1n	117.9 (14)	O1—C5—C6	117.41 (14)
C1—N2—N3	120.33 (13)	O1—C5—C4	121.55 (14)
C1—N2—H2n	114.2 (13)	C6—C5—C4	121.04 (14)
N3—N2—H2n	117.6 (13)	C5—C6—C7	119.46 (15)
C3—N3—N2	113.67 (13)	C5—C6—H6	120.3
N1—C1—N2	118.11 (14)	C7—C6—H6	120.3
N1—C1—S1	123.91 (12)	O2—C7—C6	122.00 (15)
N2—C1—S1	117.98 (12)	O2—C7—C8	117.19 (14)
N1—C2—H2A	109.5	C6—C7—C8	120.81 (14)
N1—C2—H2B	109.5	C9—C8—C7	119.10 (15)
H2A—C2—H2B	109.5	C9—C8—H8	120.4
N1—C2—H2C	109.5	C7—C8—H8	120.4
H2A—C2—H2C	109.5	C8—C9—C4	121.80 (15)
H2B—C2—H2C	109.5	C8—C9—H9	119.1
N3—C3—C4	123.08 (15)	C4—C9—H9	119.1
N3—C3—H3	118.5		
			
C1—N2—N3—C3	−176.54 (14)	C3—C4—C5—C6	−176.13 (14)
C2—N1—C1—N2	−178.35 (14)	O1—C5—C6—C7	178.45 (13)
C2—N1—C1—S1	1.2 (2)	C4—C5—C6—C7	−1.3 (2)
N3—N2—C1—N1	−15.6 (2)	C5—C6—C7—O2	−178.64 (14)
N3—N2—C1—S1	164.83 (11)	C5—C6—C7—C8	1.1 (2)
N2—N3—C3—C4	173.05 (13)	O2—C7—C8—C9	179.92 (14)
N3—C3—C4—C5	−2.2 (2)	C6—C7—C8—C9	0.2 (2)
N3—C3—C4—C9	−178.56 (14)	C7—C8—C9—C4	−1.3 (2)
C9—C4—C5—O1	−179.49 (14)	C5—C4—C9—C8	1.0 (2)
C3—C4—C5—O1	4.1 (2)	C3—C4—C9—C8	177.60 (14)
C9—C4—C5—C6	0.3 (2)		

### Hydrogen-bond geometry (Å, º)

**Table:**

*D*—H···*A*	*D*—H	H···*A*	*D*···*A*	*D*—H···*A*
O1—H1*o*···N3	0.83 (2)	1.97 (2)	2.6992 (17)	147 (2)
N1—H1*n*···N3	0.815 (19)	2.35 (2)	2.7080 (19)	107.1 (16)
O2—H2*o*···S1^i^	0.90 (2)	2.37 (2)	3.1918 (12)	152 (2)
N1—H1*n*···S1^ii^	0.815 (19)	2.763 (18)	3.3883 (13)	134.9 (17)
N2—H2*n*···O1^iii^	0.90 (2)	2.08 (2)	2.9527 (17)	162 (2)

Symmetry codes: (i) −*x*+1, −*y*+1, −*z*+2; (ii) *x*, *y*−1, *z*; (iii) *x*, *y*+1, *z*.
